# Methicillin-resistant Staphylococcus aureus isolates in a hospital of Shanghai

**DOI:** 10.18632/oncotarget.14036

**Published:** 2016-12-20

**Authors:** Xiaoguang Wang, Lin Ouyang, Lingfei Luo, Jiqian Liu, Chiping Song, Cuizhen Li, Hongjing Yan, Ping Wang

**Affiliations:** ^1^ The Center for Disease Control and Prevention of Minghang District, Minghang District, Shanghai 201101, P.R. China

**Keywords:** methicillin-resistant staphylococcus aureus (MRSA), hospital-associated MRSA, mecA gene, panton-valentine leukocidin (PVL) gene

## Abstract

Methicillin-resistant *Staphylococcus aureus* (MRSA) strains are now common both in the health care setting and in the community. Active surveillance is critical for MRSA control and prevention. Specimens of patients (200 patients with 1119 specimens) as well as medical staff and hospital setting (1000 specimens) were randomly sampled in a level 2 hospital in Shanghai from September 2011 to August 2012. Isolation, cultivation and identification of *S. aureus* were performed. Totally, 67 *S. aureus* strains were isolated. 32 *S. aureus* strains were isolated from patient samples; 13 (13/32, 40.6%) of the 32 *S. aureus* isolates were MRSA; sputum sample and patients in the department of general internal medicine were the most frequent specimen and patient group for *S. aureus* strains isolation. Remaining 35 *S. aureus* strains were isolated from the medical staff and hospital setting; 20 (20/35, 57.1%) of the 35 *S. aureus* isolates were MRSA; specimens sampled from doctors and nurses’ hands and nose and hospital facilities were the most frequent samples to isolate *S. aureus*. Resistant and virulent genes detection showed that, all 33 MRSA strains were mecA positive which accounts for 49.3% of the 67 *S. aureus* strains; 38 isolates were Panton-Valentine leukocidin (PVL) gene positive which accounts for 56.7% of the 67 *S. aureus* strains; and 17 (17/67, 25.4%) isolates are mecA and PVL genes dual positive. Multidrug-resistant strains of MRSA and PVL positive *S. aureus* are common in patients, medical staff and hospital setting, the potential health threat is worthy of our attention.

## INTRODUCTION

*Staphylococcus aureus* (*S. aureus*), a gram-positive coccal bacterium that belongs to the *Firmicutes*, was first identified in 1880 in Aberdeen, Scotland [1]. As high as 20% of the human population are long-term carriers of *S. aureus* which can be frequently found in the nose, respiratory tract, on the skin and in the lower reproductive tract of women [2]. Although *S. aureus* is an opportunistic pathogen, it is still one of the five most common causes of hospital-acquired infections and is often the cause of postsurgical wound infections [3, 4]. Infection caused by *S. aureus* range from minor skin injury to life-threatening diseases such as pneumonia, meningitis, osteomyelitis, endocarditis, toxic shock syndrome, bacteremia, and sepsis [2–6].

The formation of peptidoglycan cross-linkages provides the rigidity and strength in a bacterial cell wall [7]. During formation of the acyl-enzyme intermediate, a proton must be removed from the active site serine hydroxyl group and one must be added to the amine leaving group, in which process, DD-transpeptidase catalyzes proton transfer [8, 9]. Penicillin, an antibiotic derived from *Penicillum* fungus, can bind to the DD-transpeptidase to inhibit the enzyme's functionality and hence blocked the formation of bacterial cell walls [10, 11]. Thus, penicillin has widely adopted in clinical treatment on *S. aureus* infection since the 1940s [12]. However, some *S. aureus* produce an altered penicillin-binding protein, PBP2a, which is encoded by mecA gene carried on a large mobile genetic element called the staphylococcal chromosomal cassette mec [10–12]. The variant penicillin-binding protein binds beta-lactams with lower avidity, which results in complete resistance to all beta-lactam antibiotics including the semi-synthetic penicillins [10–13]. From the early 1970s, penicillin resistance is common in most countries and this forced the physicians finally to abandon their belief that all bacterial infections were treatable if given the vast array of effective antimicrobial agents [14].

Many penicillin-resistant *S. aureus* strains remain susceptible to penicillinase-stable penicillins such as oxacillin and methicillin [15, 16]. Historically, strains that are oxacillin and methicillin resistant were termed methicillin-resistant *S. aureus* (MRSA) [17]. In principle, MRSA strains are resistant to all ß-lactam agents, including cephalosporins and carbapenems [15–17]. In clinical practice, MRSA strains are often multiply resistant to other commonly used antimicrobial agents, including erythromycin, clindamycin, fluoroquinolones and tetracycline [18]. Therefore, MRSA always cause healthcare-associated infections and become a great challenge for clinicians [15–18]]. Carriage rates for human MRSA strains in the general population range from less than 1% to 5% [19]. Healthcare workers are expected to be at an increased risk for colonization due to occupational exposure [20]. Therefore, hospital-associated MRSA is one of the most prevalent nosocomial pathogens worldwide [21]. On the other hand, different MRSA strains with unique phenotypes have emerged in the community in recent years, which provides a reservoir of community-associated MRSA to spread [21]. Both healthcare- and community-associated MRSA strains are likely to cause life-threatening systemic infections, especially in elderly individuals and children [21].

In addition, *S. aureus* might acquire new virulence via bacteriophages, e.g. Panton-Valentine leukocidin (PVL), a group ofβ-pore-forming cytolytic toxins, could be imparted by bacteriophages [22]. Infection of *S. aureus* with PVL gene causes leukocyte destruction and necrotizing pneumonia, an aggressive condition that usually kills patients within 72 hours [22]. Therefore, active surveillance is critical for *S. aureus* control and prevention, however, the reference data of *S. aureus* surveillance from China is very limited. In this report, specimens of patients, medical staff and hospital setting were randomly sampled in a level 2 hospital in Shanghai from September 2011 to August 2012. Isolation, cultivation and identification of *S. aureus* were performed.

## RESULTS

### The distribution of bacterial isolates in clinical samples

To know the distribution of *S. aureus* isolates in clinical samples, the sputum, throat, eye and hand swabs, wound secretion, blood and urine of patients in the general internal medicine, intensive care unit, complete rooming-in, department of gerontology and department of neurosurgery were collected. Isolation, cultivation and identification of *S. aureus* were performed using these samples. Totally, 32 isolates were obtained; of the 32 *S. aureus* isolates, 17 (53.1%), 6 (18.8%), 5 (15.6%), 2 (6.3%) and 2 (6.3%) were isolated from sputum, throat, eye and hand swabs, wound secretion, blood and urine respectively (Table 1). Cefoxitin disk diffusion test showed that 13 (40.6%) of the 32 *S. aureus* isolates were MRSA; 5 (38.5%), 4 (30.8%), 2 (15.4%), 1 (7.7%) and 1 (7.7%) were isolated from sputum, throat, eye and hand swabs, wound secretion, blood and urine respectively (Table 1).

**Table 1:** The distribution of S. *aureus* isolates in clinical samples

Classification of specimens	*S. aureus* (*N* = 32)		MRSA (*N* = 13)	
	Isolates	%	Isolates	%
Sputum	17	53.1	5	38.5
Throat, eye and hand swabs	6	18.8	4	30.8
Wound secretion	5	15.6	2	15.4
Blood	2	6.3	1	7.7
Urine	2	6.3	1	7.7

MRSA, Methicillin-resistant *Staphylococcus aureus; S. aureus, Staphylococcus aureus*.

### The distribution of S. aureus carrying patients

To know the distribution of *S. aureus* isolates in clinical departments, the distribution of *S. aureus* carrying patients were calculated according to the clinical departments. Of the 32 *S. aureus* isolates, 15 (46.9%), 7 (21.9%), 4 (12.5%), 4 (12.5%) and 2 (6.3%) were isolated from the patients of general internal medicine, intensive care unit, complete rooming-in, department of gerontology and department of neurosurgery, respectively (Table 2). Of the 13 MRSA isolates, 6 (46.2%), 4 (30.8%), 1 (7.7%), 1 (7.7%) and 1 (7.7%) were isolated from patients of the general internal medicine, intensive care unit, complete rooming-in, department of gerontology and department of neurosurgery, respectively (Table 2).

**Table 2:** The distribution of S. *aureus* carrying patients

Classification of departments	S.au*reus* (*N* = 32)		MRSA (*N* = 13)	
	Isolates	%	Isolates	%
General Internal Medicine	15	46.9	6	46.2
Intensive care unit	7	21.9	4	30.8
Complete rooming-in	4	12.5	1	7.7
Department of Gerontology	4	12.5	1	7.7
Department of Neurosurgery	2	6.3	1	7.7

MRSA, Methicillin-resistant *Staphylococcus aureus; S. aureus, Staphylococcus aureus*.

### The distribution of bacterial isolates in medical staff and hospital setting

To know the distribution of *S. aureus* in diagnosis and treatment environment, swabs of doctors and nurses’ hands and nose, desk and keyboard of clinic, medical instruments, nurse's aides’ hands and nose and hospital facilities (bed, phone, door handles, faucets and toilet) were also collected for *S. aureus* isolation. Totally 35 isolated were obtained. Of the 35 *S. aureus* isolates, 10 (28.6%), 10 (28.6%), 7 (20.0%), 4 (11.4%) and 4 (11.4%) were isolated from the swabs of doctors and nurses’ hands and nose, hospital facilities (bed, phone, door handles, faucets and toilet), desk and keyboard of clinic, medical instruments, nurse's aides’ hands and nose, respectively (Table 3). Cefoxitin disk diffusion test showed that 20 (57.1%) of the 35 *S. aureus* isolates were MRSA, 5 (25.0%), 5 (25.0%), 5 (25.0%), 3 (15.0%) and 2 (10.0%) were isolated from the swabs of doctors and nurses’ hands and nose, hospital facilities (bed, phone, door handles, faucets and toilet), desk and keyboard of clinic, medical instruments, nurse's aides’ hands and nose, respectively (Table 3).

**Table 3:** The distribution of S. *aureus* isolates in medical staff and hospital setting

Classification of departments	*S. aureus* (*N* = 35)		MRSA (*N* = 20)	
	Isolates	%	Isolates	%
Doctors and nurses’ hands and nose	10	28.6	5	25.0
Hospital facilities (bed, phone, door handles, faucets and toilet)	10	28.6	5	25.0
Desk and keyboard of clinic	7	20.0	5	25.0
Medical instruments	4	11.4	3	15.0
Nurse's aides’ hands and nose	4	11.4	2	10.0

MRSA, Methicillin-resistant *Staphylococcus aureus; S. aureus, Staphylococcus aureus*.

### Resistant and virulent genes detection

**Figure 1:**
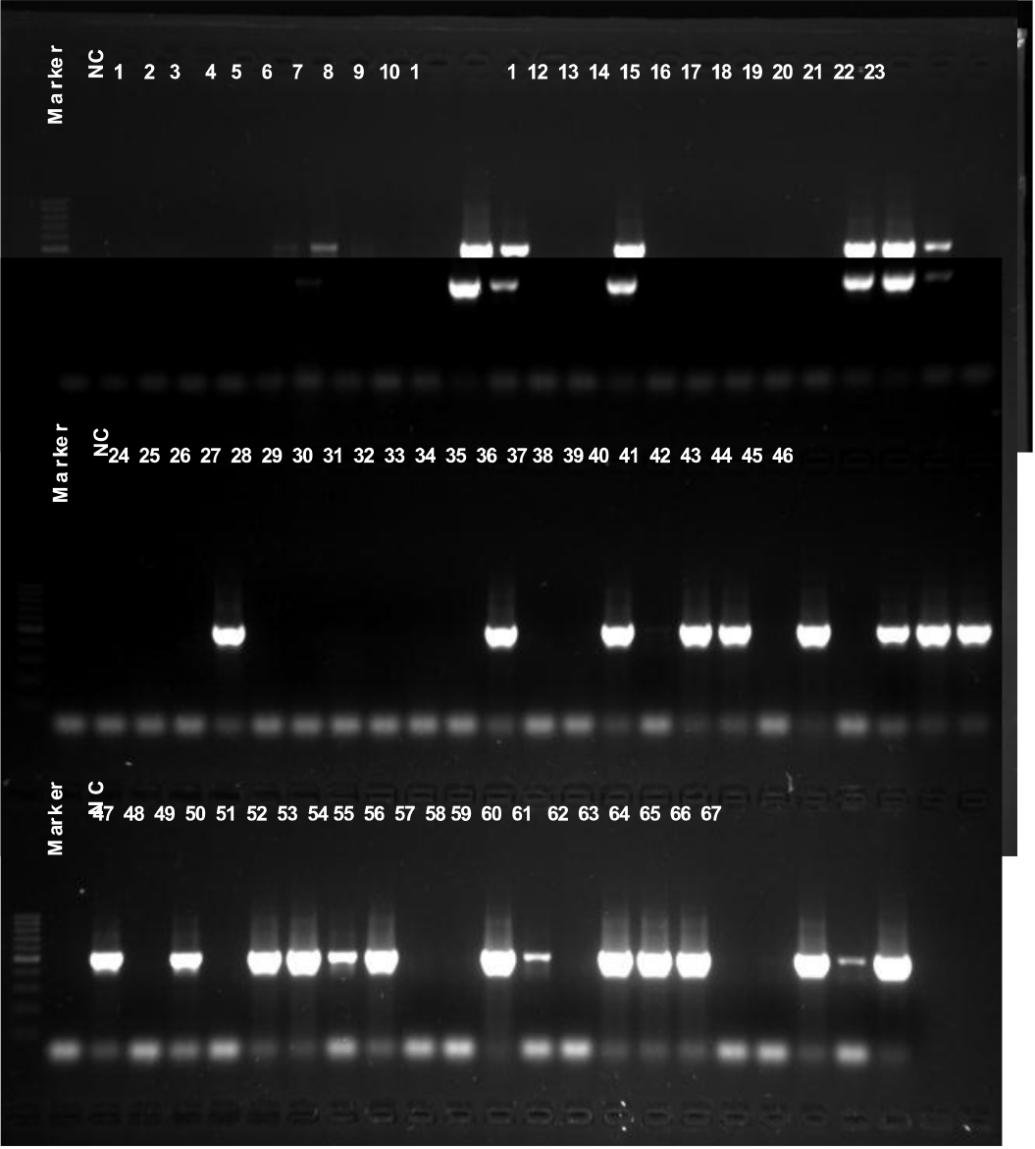
mecA gene detection All 67 isolates were submitted for mecA gene detection using PCR, a 533 bp PCR product targeting mecA gene can be seen in 1% agarose gel after 30 minutes electrophoresis. NC, negative control.

**Figure 2:**
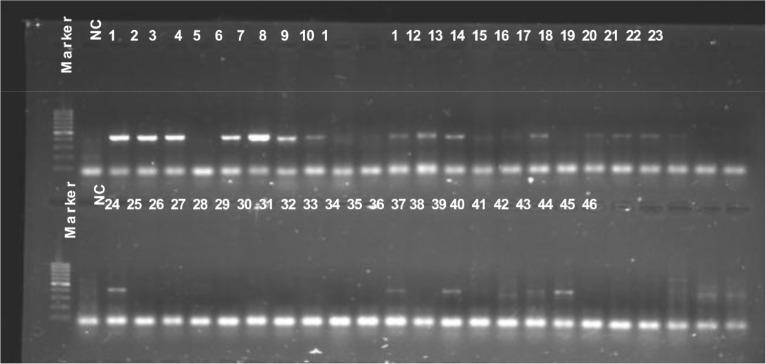
PVL gene detection All 67 isolates were submitted for PVL gene detection using PCR, a 433 bp PCR product targeting PVL gene can be seen in 1% agarose gel after 30 minutes electrophoresis. NC, negative control.

To learn the distribution of known resistant and virulent genes mecA and PVL in the isolates, all 67 *S. aureus* isolates were submitted for PCR detection using mecA or PVL targeting primers. The PCR product of mecA positive strain will be 533bp in length, as showed in Figure 1, all MRSA isolates (6, 10, 11, 14, 20, 21, 22, 27, 34, 37, 39, 40, 42, 44, 45, 46, 47, 49, 51, 52, 53, 54, 57, 58, 60, 61, 62, 65, 66 and 67), except isolates 5, 7 and 38, displayed 533 bp PCR products clearly, which suggest these isolates were mecA gene positive. The remaining three MRSA isolates, 5, 7 and 38, displayed weaker 533 bp PCR products. Different from mecA gene detection, the PVL gene positive isolates were not correlated with cefoxitin resistance. As showed in Figure 2, at least 38 isolates (1, 2, 3, 5, 6, 7, 8, 11, 12, 13, 16, 18, 19, 20, 21, 24, 34, 36, 40, 47, 48, 49, 50, 51, 52, 54, 56, 57, 58, 59, 60, 61, 62, 63, 64, 65, 66 and 67) displayed clear 433bp PLV gene fragments. 17 (6, 11, 20, 34, 40, 49, 51, 52, 54, 57, 58, 60, 61, 62, 65, 66 and 67) isolates are both mecA and PVL genes positive (Figures 1 and 2).

## DISCUSSION

*S. aureus* are successful commensal pathogens in our body because they can adapt to selective pressures (e.g. use of antibiotics) rapidly via a number of mechanisms [22]. Among these mechanisms, mobile genetic elements (MGEs) play a central role in this adaptation process, because MGEs are a means to transfer genetic information among and within bacterial species and MGEs encode putative virulence factors that confer resistance to antibiotics [22]. MGEs mainly consist of chromosome cassettes, insertion sequences, transposons, pathogenicity islands, plasmids, phages and so on [22]. Staphylococcal cassette chromosomes (SCCs) are relatively large fragments of DNA that always insert into the *orf*X gene on the *S. aureus* chromosome [22]. SCCs of MRSA encode the methicillin resistance gene (mecA), therefore, MRSA poses a great threat to the control of clinical infection of *S. aureus* [15–18]. At present, in the case of limited effective antibiotics, active monitoring is the important means of MRSA control and prevention. In this report, to learn the distribution of MRSA in local hospital setting, specimens of patients (200 patients with 1119 specimens) as well as medical staff and hospital setting (1000 specimens) were randomly sampled in a level 2 hospital in Shanghai from September 2011 to August 2012. Totally, 67 *S. aureus* strains were isolated and 33 (49.3%) were MRSA. Of the 67 *S. aureus* strains, 32 were isolated from patient samples; 13 (40.6%) of the 32 *S. aureus* isolates were MRSA; sputum and patients of general internal medicine were the most frequent specimen and patient group for *S. aureus* strains isolation respectively. Remaining 35 *S. aureus* strains were isolated from the medical staff and hospital setting; 20 (57.1%) of the 35 *S. aureus* isolates were MRSA; specimens sampled from doctors and nurses’ hands and nose and hospital facilities were the most frequent specimen to isolate *S. aureus*. Although the detection rate of MRSA is lower than most reports [15–18], MRSA is widely prevalent in our patients and hospital setting. Thus, the potential health threat of MRSA is worthy of our attention.

The role of the PVL gene in virulence is still in dispute, some studies showed that presence of PVL is associated with increased virulence of certain strains of *S. aureus* [23, 24]. Infection of *S. aureus* with PVL causes leukocyte destruction and necrotizing pneumonia, an aggressive condition that usually kills patients within 72 hours [19, 21]. On the contrary, a recent systematic evaluation showed that PVL gene had not increased virulence of community-associated MRSA [25]. Even so, surveillance of virulent genes has the same importance as MRSA monitoring. In this study, 38 isolates were PVL gene positive and 17 (17/67, 25.4%) isolates were mecA and PVL genes dual positive. Our results indicate that the potential public health threat caused by PVL positive *S. aureus* infection can not be ignored.

According to the popular locations, MRSA was divided into community-associated MRSA and hospital-associated MRSA, the genovariation, virulence and epidemic characteristics differed between community-associated MRSA and hospital-associated MRSA [21]. In this report, we had only focused on the hospital-associated MRSA, our data showed that MRSA and PVL positive *S. aureus* are common both in patients and diagnosis and treatment environment. In the future, we will continue to study the clinical features of MRSA infection and the epidemic characteristics of community-associated MRSA.

## MATERIALS AND METHODS

### Samples

To know the distribution of *S. aureus* in clinical samples, the sputum, throat, eye and hand swabs, wound secretion, blood and urine of patients in the general internal medicine, intensive care unit, complete rooming-in, department of gerontology and department of neurosurgery were collected in a local level 2 hospital of Shanghai (There are more than 300 hospitals distributed in the 16 administrative districts in Shanghai, the studied hospital is a center hospital of one district with bed number more than 1,000) from September 2011 to August 2012. The Random sampling rate for patients was 1:15. Finally, 1119 specimens from 200 patients were collected. To know the distribution of *S. aureus* in medical staff and hospital setting, 1000 swabs sampled from doctors and nurses’ hands and nose, desk and keyboard of clinic, medical instruments, nurse's aides’ hands and nose and hospital facilities (bed, phone, door handles, faucets and toilet) were also collected at a random sampling rate of 1:10. Finally, 2119 samples were collected and applied for *S. aureus* isolation and culture.

This study was carried out following the rules of the World Medical Association Declaration of Helsinki and approved by the Internal Review Board at the Centers for Disease Control and Prevention of Shanghai. Written informed consent was obtained according to the guidelines of the National Ethics Regulation Committee. Participants, immediate relatives, caregivers, or legal guardians informed the participants of their right to withdraw consent.

### Isolation, cultivation and identification of *S. aureus*

Specimens were inoculated in the 7.5% NaCl broth and cultured at 36°C for 18 hours, then, the primary cultures were transferred into CHROMagar plate (CHROMagar, Paris, France) and continue to culture at 36°C for 18–24 hours. Identification of *S. aureus* was performed by using the following tests according to the Clinical and Laboratory Standards Institute (CLSI) of United States recommendations (M100-S24, Performance Standards for Antimicrobial Susceptibility Testing; Twenty-Fourth Informational Supplement): morphology following Gram staining, catalase activity, mannitol fermentation, gelatin hydrolysis, mixed sugar fermentation by methyl red test, Voges–Proskauer test for acetoin production, coagulase activity and β-haemolysis.

### Cefoxitin disk diffusion test

MRSA was identified by following the recommendations of the CLSI. For methods in detail, see CLSI Approved Standard M100-S24 (M100-S24, Performance Standards for Antimicrobial Susceptibility Testing; Twenty-Fourth Informational Supplement). Zone diameter in disk diffusion test ≥ 22 mm was defined as cefoxitin susceptible; Zone diameter in disk diffusion test ≤ 21 mm was defined as MRSA. *S. aureus* strains ATCC25923 (http://www.atcc.org/Products/All/25923.aspx); ATCC43300 (http://www.atcc.org/Products/All/43300.aspx); ATCC25922 (http://www.atcc.org/Products/All/25922.aspx) and ATCC27853 (http://www.atcc.org/Products/All/27853.aspx) were used as reference strains.

### Resistant and virulent genes detection

The known resistant and virulent genes, mecA located in the staphylococcal chromosome cassette mec and Panton-Valentine leukocidin (PVL) gene, were detected using polymerase chain reaction (PCR). For mecA, a 533 bp PCR product (NCBI Reference Sequence: Staphylococcus aureus TN/CN/1/12 mecA gene for PBP2a family beta-lactam-resistant peptidoglycan transpeptidase MecA, complete cds. GenBank: NG_047945.1) will be obtained using the following primers: forward, 5′AAAATC GATGGTAAAGGTTGGC3′; reverse, 5′AGTTCTGCA GTACCGGATTTGC3′. For PVL, a 433bp PCR product (NCBI Reference Sequence: Staphylococcus phage PVL-Sa2GN3 proviral DNA, lukS-PV, lukF-PV genes, complete cds. GenBank: LC086375.1) will be obtained using the following primers: forward, 5′ATCATTAGGT AAAATGTCTGGACATGATCCA3′; reverse, 5′GCATC AAGTGTATTGGATAGCAAAAGC3′). All PCR was performed using a 2 x Direct PCR mix (Biovisualab, Shanghai, China), the 2 x Direct PCR mix can detect bacteria DNA without nucleic acid extraction and purification. PCR temperature cycles were: 95°C 3 min; 30 cycles of 95°C 30 seconds, 55°C 30 seconds and 72°C 30 seconds. The correct PCR products were confirmed by 30 minutes electrophoresis in 1% agarose gel.
